# Repeat application of microneedles does not alter skin appearance or barrier function and causes no measurable disturbance of serum biomarkers of infection, inflammation or immunity in mice *in vivo*

**DOI:** 10.1016/j.ejpb.2017.04.029

**Published:** 2017-08

**Authors:** Eva M. Vicente-Perez, Eneko Larrañeta, Maelíosa T.C. McCrudden, Adrien Kissenpfennig, Shauna Hegarty, Helen O. McCarthy, Ryan F. Donnelly

**Affiliations:** a School of Pharmacy, Queen’s University Belfast, 97 Lisburn Road, Belfast BT9 7BL, UK; b The Wellcome-Wolfson Building, Centre for Experimental Medicine, School of Medicine, Dentistry and Biomedical Science, Queen's University Belfast, 97 Lisburn Road, Belfast BT9 7BL, UK; c Royal Victoria Hospital, 274 Grosvenor Road, Belfast BT12 6BA, UK

**Keywords:** Microneedles, Repeat application, Skin barrier function, Biomarkers, Regulatory

## Abstract

We address, for the first time, the impact of skin insertion on multiple occasions of polymeric microneedle arrays in an animal model *in vivo*. Dissolving microneedle arrays prepared from aqueous blends of 20% w/w Gantrez® S-97 BF and 40% w/w poly(vinyl pyrrolidone) 58 kDa and hydrogel-forming microneedle arrays prepared from aqueous blends of and poly(ethyleneglycol) 10 kDa were repeatedly applied to the skin of hairless mice *in vivo*. Skin appearance and skin barrier function, as illustrated by measurement of transepidermal water loss, were not measurably altered during the entire study period. Biomarkers of infection, immunity and inflammation/irritation were also statistically unchanged, regardless of the microneedle formulation, needle density or number of applications. Mice remained healthy throughout and continued to gain weight during the study. For example, transepidermal water loss values were typically in the range 10–15 g m^−2^ h^−1^ immediately prior to microneedle insertion and 15–25 g m^−2^ h^−1^ immediately following microneedle removal, regardless of when they were measured during the study periods. Serum biomarker levels, measured immediately post-mortem were always in the range 10–20 µg ml^−1^ for C-reactive protein, 0.5–1.5 mg ml^−1^ for Immunoglobulin G and 1000–2500 pg ml^−1^ for interleukin 1-β and were never statistically different from untreated controls. No measurable levels of tumour necrosis factor-α were found in any animals. These findings are encouraging for the formulations investigated, suggesting that their repeated use by patients will not cause undesirable side-effects. By beginning to address potential regulatory questions at an early stage, the microneedles field will be ideally-placed to take advantage of the potential market. This work illustrates a potential pre-clinical strategy for development of regulatory dossiers on microneedle technologies.

## Graphical abstract

**Figure:**
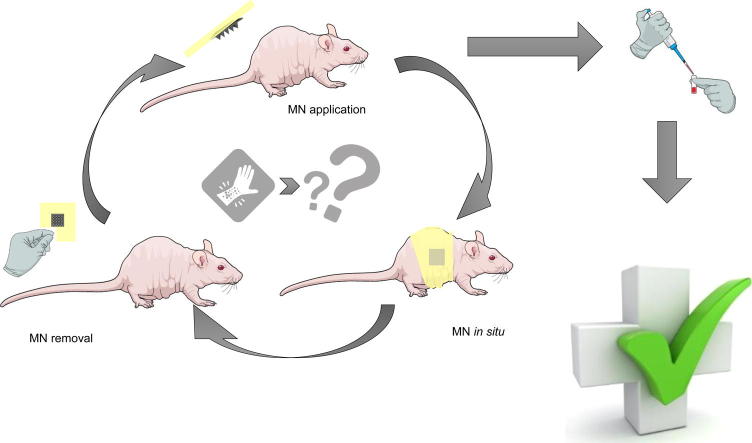

## 1 Introduction

Microneedle arrays (MN), minimally-invasive devices that painlessly and without drawing blood penetrate the skin’s *stratum corneum* barrier to greatly expand the range of substances that can be delivered into and across the skin have shown tremendous promise over recent years. However, no true MN array-based product is currently marketed. Indeed, bringing a new drug or a new dosage form to market is a costly and time-consuming process. Increasing regional and international regulation, while necessary, has only served to augment costs [1]. Guidance and regulations established by the US Food and Drug Administration (FDA) has played a critical role in providing a framework for many regulatory bodies worldwide [2], [3]. During the development of a new drug or medical device, an assessment of potential biological risks must be performed. While the possible regulatory questions pertaining to various MN designs have been speculated upon in the literature [4], [5], [6], [7], only a few relevant experimental studies have been published. Such investigations, using a mixture of animal models and human volunteers, recorded changes in skin barrier function through transepidermal water loss (TEWL) measurements [8], [9], local skin irritation and subcutaneous blood flow [8], [9], [10], [11], dimensions of the induced skin pores [12], [13], [14], histological changes and inflammatory cell infiltrations [12], [15].

Safety studies performed to date have assumed that MN will always be used for their most commonly suggested application; as vaccine delivery devices. Accordingly, measurements were taken after a single MN array insertion. However, our own work has shown that appropriately-designed MN patches are capable of delivering therapeutically relevant doses of drugs transdermally, even when such drugs are not of high potency [16], [17]. We have also illustrated that MN may have a role to play in minimally-invasive patient monitoring [18], [19]. In both of these latter applications, MN will certainly be inserted into the skin on a regular basis, with daily use not out of the question. Indeed, even MN vaccines are likely to be inserted more than once, given the need for prime and boost regimes to attain strong immunisation for most current vaccines.

It is our considered view that dissolving polymeric MN arrays will find their most appropriate use as vaccine delivery vehicles, due to their ordinarily-limited loading capacity and their tendency to deposit polymer in skin. As such they will be inserted in skin a relatively small number of times over a patient’s lifetime. In contrast, our hydrogel-forming MN, which swell in skin to allow controlled delivery of a drug substance from an attached patch-type drug reservoir, or capture skin interstitial fluid for use in patient monitoring, are removed intact from skin, depositing no measurable amount of polymer [16], [17], [18], [19]. Since patients may need a drug administered on a daily basis or routine monitoring for a chronic condition, these MN are likely to be inserted into skin on a much more regular basis. Since regulators will undoubtedly ask questions about the impact of repeated MN insertion, we designed a series of pre-clinical experiments to investigate this, the results of which are detailed here.

## 2 Materials and methods

### 2.1 Chemicals

Gantrez® S-97 BF, a copolymer of methyl-vinyl ether-co-maleic-acid (PMVE/MA), M_w_ = 1,500,000 Da) and poly(vinyl pyrrolidone) (PVP, M_w_ = 58,000 Da) were provided by Ashland, Surrey, UK. Polyethylene glycol (PEG, M_w_ = 10,000 Da) was obtained from Sigma-Aldrich, Steinheim, Germany. Isoflurane was acquired from Abbott Laboratories, Illinois, USA. Formaldehyde-saline was purchased from TCS Biosciences Ltd, Buckingham, UK. All other chemicals used were of analytical reagent grade.

### 2.2 Animals

Crl: SKH1-Hr^hr^ mice were chosen as the test animals because they have been previously used as a dermatological animal model due to their lack of hair [20]. This characteristic avoided the continuous hair removal procedure prior to MN insertion that could potentially irritate skin. Importantly, unlike other strains of hairless mice, Crl: SKH1-Hr^hr^ are immunocompetent [21].

All animal experiments throughout this study were approved by the Ethics Committee of the QUB Biological Services Unit and conducted according to the policy of the Federation of European Laboratory Animal Science Associations (FELASA) and The European Convention for the protection of Vertebrate Animals used for Experimental and Other Scientific Purposes, with implementation of the principle of the 3Rs (replacement, reduction, refinement). Any animals with 20% weight loss during the study were to be removed for euthanasia, but this did not occur. No skin reactions to MN occurred either. At the end of the experiment, euthanasia was by carbon dioxide.

### 2.3 Biomarker assays

Four biomarkers were measured in mice sera at the end of the experiment. The biomarkers chosen were: C-reactive protein (CRP), interleukin 1-β (IL-1β), tumour necrosis factor-α (TNF-α) and immunoglobulin G (Ig G). CRP levels rapidly increase within hours after tissue injury or infection, suggesting that it contributes to host defence and that it is part of the innate immune response [22]. CRP is a biomarker typically used to diagnose infection and sepsis [23], [24]. CRP serum content was quantified to confirm that repeated MN skin insertion does not promote skin or systemic infection. TNF-α is considered the principal mediator of events involved in inflammation and immunity, playing a preponderant role in defence against infections by fungi, bacteria and even parasites [25]. TNF-α also seems to be a key regulator of local T-cell proliferation and subsequent development in autoimmune disorders of the skin [26]. IL-1β is one of the key members of the IL-1-like family of cytokines. This family consists of 11 proteins that regulate inflammation caused by bacterial or viral infections or in response to molecular patterns associated with danger and damage. The main producers of IL-1β are cells of the innate immune system; monocytes and macrophages. Other cell types, however, such as epithelial cells, astrocytes, and fibroblasts, present in the skin are also able to express IL-1β [27], [28]. Levels of IL-1β have been connected with inflammatory processes in cancer and skin psoriasis [29], [30]. Ig G is principally responsible for the recognition, neutralization; and elimination of pathogens and toxic antigens [31]. Ig G is produced in a delayed response to an infection and can be retained in the body for prolonged periods of time.

Mouse DuoSet ELISA quantification kits for IL-1β, CRP and TNF-α were purchased from R&D Systems Inc., Minneapolis, USA. Mouse IgG Total ELISA Ready-SET-Go! was acquired from Affymetrix, California, USA. In all cases, appropriate dilutions of samples were made so as to align with the relevant calibration ranges.

### 2.4 Microneedles

Laser-engineered silicone micromoulds were manufactured using a previously detailed method [32]. The features of the needles produced were conical shapes with: 600 μm heights, 300 μm base widths and 150 μm needle interspacing on a 0.5 cm^2^ area (19 × 19 design) and 600 μm heights, 300 μm base widths and 300 μm needle interspacing a 0.5 cm^2^ area (11 × 11 design).

**Fig. 1:**
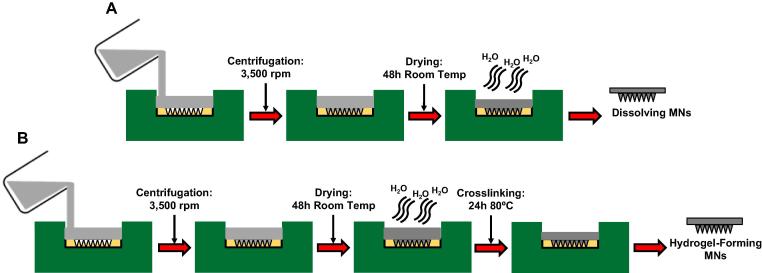
Diagram A represents dissolving MN array manufacture. Hydrogel-forming MN array manufacture is represented in diagram B.

As shown in Fig. 1, hydrogel-forming MN were prepared from aqueous blends of 15% w/w PMVE/MA and 7.5% w/w PEG as previously described [16], [17], [18], [19]. Dissolving MN were prepared from aqueous blends of 20% w/w PMVE/MA and 40% w/w of PVP, respectively. In each case, formulations were first centrifuged at 3500 rpm for 15 min to ensure a homogenous blend and to remove any air bubbles. An aliquot of 0.5 g was then cast into the silicone micromould and centrifuged again at 3500 rpm for a further 15 min. Following air drying, hydrogel-forming MN were crosslinked by esterification. In all cases, the sidewalls formed during micromoulding were removed using a heated scalpel prior to use.

### 2.5 *In vivo* multiple application of polymeric microneedle arrays

A total number of 36 healthy hairless mice, 18 males and 18 females, were used. Animals were housed according to gender, polymer, and needle density. There were 12 groups, with three mice in each group. Two control groups of mice had no MN inserted into their skin at any time, while the remaining groups had one of the two densities of either dissolving of hydrogel-forming MN arrays inserted into their skin repeatedly. Mice always had the same type of MN inserted and there was no interchanging once a mouse was assigned to a particular group. MN were always applied at the same approximate sites on each mouse. Treated MN mice were compared with negative control groups (only surgical tape applied) to verify if any significant changes were observed in the skin or serum biomarkers.

**Fig. 2:**
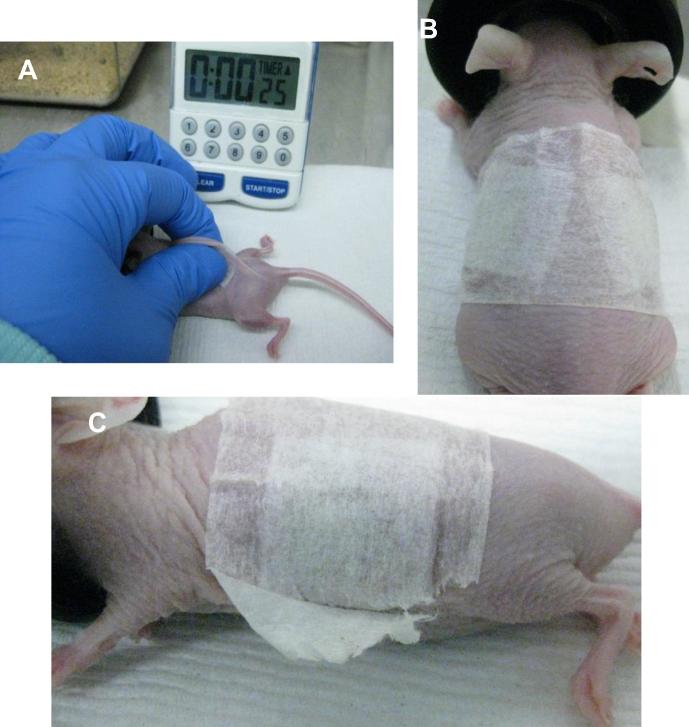
MN array skin insertion procedure (A). MN insertion set-up immediately after application with two MN inserted at opposite positions on the lower back of the mouse (B). Insertion site of a male mouse preceding MN array removal (C).

**Fig. 3:**
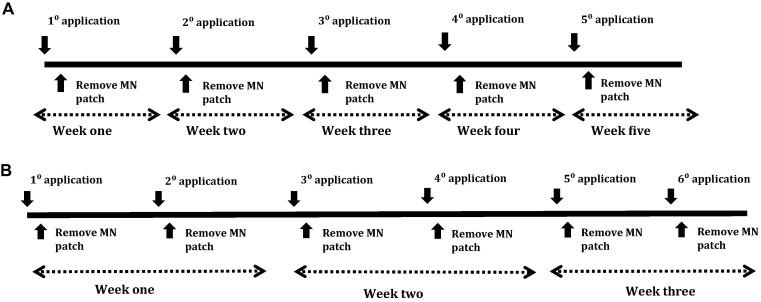
Diagram A describes the dissolving MN array application and removal regime. The hydrogel-forming MN patch application and removal regime is detailed in diagram B.

Dissolving MN arrays were inserted once per week for five consecutive weeks in each hairless mouse. In each case, two MN arrays were inserted into two opposite positions in skin of the lower back (Fig. 2). MN arrays were inserted by pinching the skin application site for 30 s with the thumb and forefinger. After MN insertion, arrays were kept in place for 24 h using surgical tape (Micropore™, 3M, Bracknell, UK). The application protocol for hydrogel-forming MN arrays was the same. However, the frequency of insertion was increased to twice per week during three successive weeks. The insertion/application protocols are summarised in Fig. 3.

Mouse well-being was monitored by recording weight once per week during the performance of the study. Skin appearance was tracked over time *via* digital pictures (Nikon Coolpix™, Nikon, Tokyo, Japan) taken under controlled lighting conditions. Photographs of application site were taken before MN patch insertion and after removal in each case, so as to track any possible skin changes.

Transepidermal water loss (TEWL) measurements were taken as a measure of skin barrier function state (VapoMeter®, Delfin Technologies Ltd, Kuopio, Finland). Measurements were taken directly from the two application sites immediately before each MN insertion and again immediately following each MN removal.

### 2.6 Sacrifice, blood collection and biomarker quantification

Mice were euthanised immediately after the last MN array was removed. Subsequently, blood was collected *via* cardiac puncture. To gather serum samples for the ELISAs used to quantify the biomarkers, Eppendorf tubes were incubated at 37 °C for 45 min. Afterwards, tubes were centrifuged at 4 °C at 200 rpm for 15 min. Supernatant was carefully collected and aliquoted to be stored at −80 °C prior to assay by ELISA, following the manufacturers’ instructions and measuring absorbances using an Enspire® platereader (Perkin-Elmer, London, UK) at the appropriate wavelengths.

### 2.7 Statistical analysis

Data was analysed, as appropriate, using the Mann-Whitney test, Kruskal-Wallis test and Dunns post-test to compare control group and MN-treated groups. In all cases, *p* < 0.05 was considered as a significant difference.

## 3 Results

### 3.1 Fabrication of polymeric microneedle arrays and *in vivo* application and removal

**Fig. 4:**
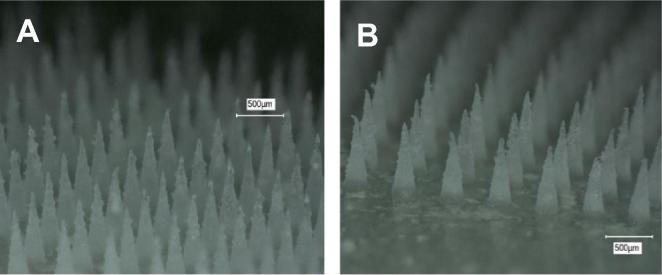
Micrographs exemplify the sharp tips of a 58 kDa PVP 19 × 19 MN array (A) and of an 11 × 11 MN array prepared from Gantrez® S-97 (B).

**Fig. 5:**
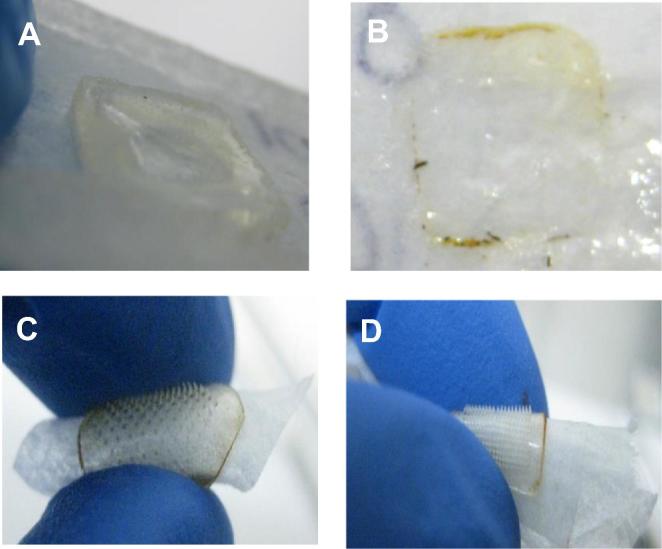
Dissolving 58 kDa PVP MN arrays dissolved after 24 hours’ skin insertion in mouse skin *in vivo* (A) and dissolved Gantrez® S-97 MN arrays after 24 hours’ skin insertion in mouse skin *in vivo* (B). Hydrogel-forming MN arrays after removal following 24 hours’ skin insertion in mouse skin *in vivo* displayed flexibility due to the uptake of interstitial fluid regardless of whether the needle density used was 11 × 11 (C) or 19 × 19 (D).

A total number of 384 MN arrays were produced using the three formulations and two needle densities. As shown in Fig.4A and B, MN arrays had, in each case, fully-formed needles with sharp tips. As a normal behaviour, mice were inclined to eat the surgical tape around the abdominal area. However, MN patches were still kept in place and always remained inserted until removal after 24 h, as shown in Fig.5C and D.

A total of 120 dissolving MN arrays were inserted into mouse skin *in vivo* over a 5-week period. Upon removal after 24 hours’ insertion, the needles had universally dissolved in skin, leaving only a gel-like baseplate (Fig. 5A and B). In total, 140 hydrogel-forming MN patches inserted into mouse skin *in vivo* over a 3-week period. All needles remained fully intact when recovered after 24 hours’ insertion. The hydrogel-forming MN arrays had swollen in skin and were now flexible in nature (Fig. 5C and D).

### 3.2 *In vivo* transdermal water loss measurements, monitoring of skin appearance and mouse wellbeing

**Fig. 6:**
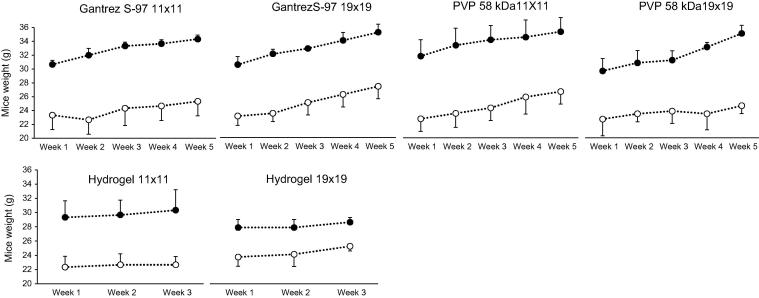
Graphs display the recorded weights of the mice over the entire experimental period. Each graph represents one MN-treated group with a particular formulation and needle configuration. Female -○- and male -●- values were plotted separately (Means ± S.D.; n = 3).

Animal welfare was assessed through the observation of animal behaviour and monitoring of animal weight. Fig. 6 illustrates the weight monitoring graphs for the duration of the experiments. Weights were recorded at the beginning of each week. Each graph represents a MN array type and a needle density. Additionally, female and male weight records were plotted separately. It can observed from the graphs that mouse weight increased over time, indicating that all animals remained healthy.

Skin appearance was surveyed over the entire duration of the study. Pictures of the application area were taken before each and every MN insertion and after each and every MN removal. Mice were individually identified by ear punch; no ear punch left ear punch and right ear punch. Mouse identification allowed faithful recording and comparison of skin pictures, TEWL measurements and biomarkers values for each mouse.

**Fig. 7:**
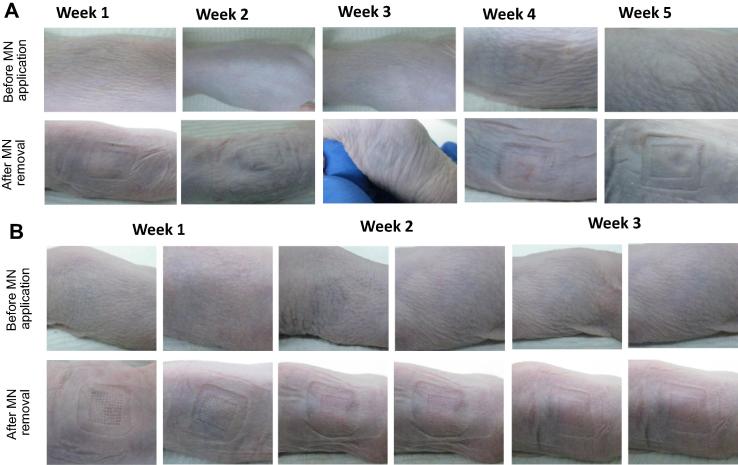
Photographs were taken to monitor skin appearance over the entire duration of the experimental period. Exemplar images are presented here. Images of “no ear punch” male to which 19 × 19 dissolving PMVE/MA MN arrays (A) and “no ear punch” female to which 11 × 11 hydrogel-forming MN arrays (B) were applied.

In general, immediately after MN removal, all mice displayed mild erythema at the MN application site. This is most likely due to MN insertion into skin and surgical tape removal. Indeed, some control mice showed mild and short-lived erythema around the site of tape removal. Importantly, erythema had universally and completely dissipated prior to the next MN insertion in the test animals. No permanent skin appearance changes were noted for mice to which dissolving MN were inserted, regardless of MN formulation or density (2 arrays per application per week over a period of 5 consecutive weeks), as shown in Fig.7A. The hydrogel-forming MN-treated group did not show any notable skin alteration, even when MN arrays were inserted twice weekly over 3 weeks. Increased frequency compared to the dissolving MN did not affect external skin appearance, as Fig.7B illustrates.

**Fig. 8:**
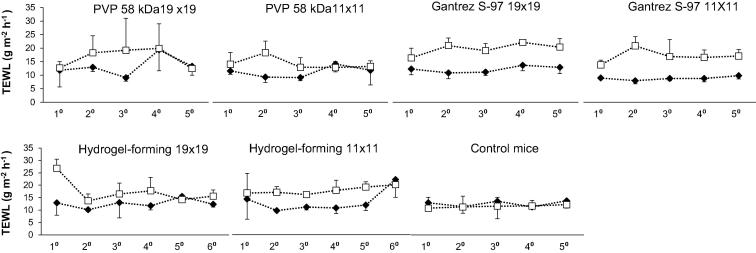
Recorded transepidermal water loss values immediately before MN insertion -♦- and immediately after MN removal -□- from the skin of mice over the entire experimental period. Each graph represents one entire MN-treated group with a particular formulation and needle configuration (Means ± S.D.; n = 6).

TEWL results are illustrated in Fig. 8. Statistical analyses were carried out comparing TEWL values for female and male mice. It was verified that there was no statistical differences between genders. Therefore, TEWL values were plotted on the same graphs. This part of the study explored skin barrier function re-establishment after it had been compromised in a repeated manner using high-density polymeric MN patches. In each case, TEWL was elevated immediately following MN removal, but had returned to baseline prior to the next scheduled MN insertion, with no statistically-significant trends identified.

### 3.3 Measurement of inflammation, irritancy and immunological reaction biomarkers in mouse sera

**Fig. 9:**
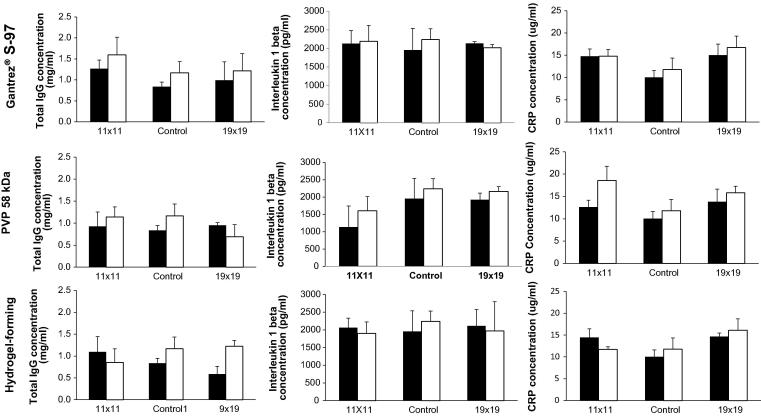
Serum biomarker levels of each MN-treated group. Values for female (white bars) and male (black bars) mice were plotted separately (Means ± S.D.; n = 3).

Encouragingly, TNF-α levels were undetectable in all mice. Data for the other biomarkers is presented in Fig. 9. No statistical differences were seen between the measured biomarkers levels compared to one another or control, regardless of formulation type, needle density, number of applications or mouse gender (*p* > 0.05 in all cases). These analyses suggest that repeated insertions of dissolving or hydrogel-forming MN arrays do not stimulate the humoral immune system, do not produce sufficient trauma to elevate circulating blood levels of IL-1β and do not caused infection or trigger an inflammatory response cascade.

## 4 Discussion

By definition, MN penetrate the skin’s protection *stratum corneum* barrier and enter the viable epidermis and, in many cases the dermis. These areas of the body are ordinarily sterile and MN are not ordinarily manufactured as sterile products. Nevertheless, only rare and isolated examples of adverse events exist and inappropriate use was the cause in the documented cases [7]. MN insertion typically reduces skin barrier function, as evidenced by increases in transepidermal water loss and causes local inflammation and/or irritation to the skin, illustrated by erythema and oedema [8], [15], [33], [34], [35]. However, these phenomena typically resolve within a period of several hours, with no long lasting effects reported [8], [15], [33], [34], [35]. Skin recovery post MN removal is likely to involve inflammatory and lipid production processes, with prostaglandin release [34] and cholesterol synthesis both possibly important [36]. High speed application of MN has been shown to cause localised death of viable cells in skin [37]. This boosts immune response to delivered vaccine antigens, but is likely to be undesirable if the MN are not intended to be used in vaccination, especially if they are to be inserted in skin regularly. These phenomena, whilst unlikely to cause prolonged adverse events, are likely to raise questions from regulatory bodies. In spite of this, most published work on MN focuses on their design and use, rather than specific safety evaluations or regulatory aspects of translation to clinic. However, it is vitally-important that any questions are addressed at an early stage, so as not to slow progression to patient benefit and commercial return.

We expect any approved MN products to be inserted into the skin of the same patient on more than one occasion, regardless of the therapeutic indication. Indeed, Zosano Pharma [38] and Corium [39] have conducted extensive clinical studies, up to completion of Phase II, where coated titanium and dissolving polymeric MN, respectively, were repeatedly inserted into the skin of post-menopausal women over a prolonged period of time for delivery of parathyroid hormone analogue. No adverse events were reported, though no data on skin barrier function or serum biomarkers was published in either case. We believe that it will be important to show regulators that repeated insertion of MN into skin does not disturb skin barrier function, or yield any undesirable systemic effects. This must clearly first be done in an animal model, prior to human studies and should use non-drug-loaded MN to illustrate the effect of the MN material, MN density and number of applications on the measured parameters. Further studies would then clearly need to be done as a follow-on where drug(s) to be delivered are included in the formulations.

In the present study, no significant changes to skin appearance or skin barrier function were observed, regardless of the MN formulation, needle density or number of insertions. Serum biomarkers of irritation/inflammation, infection and immunity were not significantly disturbed by the end of the study periods. These findings are encouraging for the formulations investigated, suggesting that their repeated use by patients will not cause undesirable side-effects.

## 5 Conclusion

Commercialisation will be the primary route by which patients will benefit from MN technology. In addressing potential regulatory questions at an early stage, the field will be ideally-placed to take advantage of the potential market. This study addressed, for the first time, the insertion on multiple occasions of polymeric MN patches *in vivo*. This may ultimately reflect the normal pattern of use of approved MN products. Since skin barrier function was not compromised during the study and measurement of biomarkers of immunity, irritation/inflammation and infection did not raise any concerns, we are now preparing a follow-on study with human volunteers using MN manufactured to GMP standards. The outcomes of such work will allow us to develop a regulatory dossier to support future applications for marketing approval.
