# Corrigendum to “Repeat application of microneedles does not alter skin appearance or barrier function and causes no measurable disturbance of serum biomarkers of infection, inflammation or immunity in mice in vivo” [Eur. J. Pharm. Biopharm. 117 (2017) 400–407]

**DOI:** 10.1016/j.ejpb.2025.114926

**Published:** 2026-01

**Authors:** Eva M. Vicente-Perez, Eneko Larrañeta, Maelíosa T.C. McCrudden, Adrien Kissenpfennig, Shauna Hegarty, Helen O. McCarthy, Ryan F. Donnelly

**Affiliations:** a School of Pharmacy, Queen’s University Belfast, Medical Biology Centre, 97 Lisburn Road, Belfast BT9 7BL, UK; b The Wellcome-Wolfson Building, Centre for Experimental Medicine, School of Medicine, Dentistry and Biomedical Science, Queen’s University Belfast, 97 Lisburn Road, Belfast BT9 7BL, UK; c Royal Victoria Hospital, 274 Grosvenor Road, Belfast BT12 6BA, UK

The authors regret that an error occurred during the assembly of Figure 7B in the originally published version of this article. Specifically, the panels for Week 2 and Week 3 contain duplicated images. Unfortunately, the original images are no longer available for correction. However, the purpose of the figure was to demonstrate that the polymeric microneedle-treated group showed no notable skin alterations, even when microneedle arrays were applied twice weekly over a three-week period. We believe this observation remains well-supported by the data presented. In addition, trans-epidermal water loss (TEWL) measurements, along with biomarkers of inflammation, irritation, and immunological response, were assessed to evaluate the impact of microneedle application. These results, which we have subsequently confirmed in both humans and minipigs [1,2], demonstrate the safety of repeat application of polymeric microneedles. We apologise for any inconvenience this may have caused.
